# Modulation of iTBS after-effects via concurrent directional TDCS: A proof of principle study

**DOI:** 10.1016/j.brs.2017.03.009

**Published:** 2017

**Authors:** Sara Tremblay, Ricci Hannah, Vishal Rawji, John C. Rothwell

**Affiliations:** Sobell Department of Motor Neuroscience and Movement Disorders, UCL Institute of Neurology, London, UK

**Keywords:** Transcranial magnetic stimulation, Intermittent theta burst stimulation, Transcranial direct current stimulation, Primary motor cortex, AP, anterior-posterior, iTBS, intermittent theta burst stimulation, M1, primary motor cortex, MEP, motor evoked potential, PA, posterior-anterior, TDCS, transcranial direct current stimulation

## Abstract

**Background:**

Polarising currents can modulate membrane potentials in animals, affecting the after-effect of theta burst stimulation (TBS) on synaptic strength.

**Objective:**

We examined whether a similar phenomenon could also be observed in human motor cortex (M1) using transcranial direct current stimulation (TDCS) during monophasic intermittent TBS (iTBS).

**Methods:**

TDCS was applied during posterior-anterior iTBS using three different conditions: posterior-anterior TDCS (anode 3.5 cm posterior to M1, cathode 3.5 cm anterior to M1), anterior-posterior TDCS (cathode 3.5 cm posterior to M1, anode 3.5 cm anterior to M1), and sham TDCS.

**Results:**

When the direction of TDCS (posterior-anterior) matched the direction of the electrical field induced by iTBS, we found a 19% non-significant increase in excitability changes in comparison with iTBS combined with sham TDCS. When the TDCS was reversed (anterior-posterior), the excitatory effect of iTBS was abolished.

**Conclusion:**

Our findings suggest that excitatory after-effects of iTBS can be modulated by directionally-specific TDCS.

## Highlights

• M1 excitability can be modulated by concurrent TDCS and iTBS.
• Posterior-anterior TDCS during iTBS induces a qualitative increase in M1 iTBS after-effects.
• Anterior-posterior TDCS during iTBS abolishes the after-effects of iTBS over M1.

## Background

The hand area of M1 is sensitive to the direction of electrical current induced by TMS; current flowing in a posterior-anterior (PA) direction across the central sulcus elicits motor evoked potentials (MEPs) in hand muscles at lower intensities and shorter latencies than anterior-posterior (AP) induced currents [1], [2], [3]. We recently reported that changes in M1 excitability produced by transcranial direct current stimulation (TDCS) are also influenced by current direction. TDCS delivered with the anode posterior and the cathode anterior to the M1 hotspot produces larger after-effects than other directions [4].

Intermittent theta burst stimulation (iTBS) is typically applied using biphasic stimulus pulses which induce an initial PA current across M1. It produces a moderate but highly variable facilitation of motor evoked potentials (MEPs) (potentiation of ∼20–30% and ∼50% of responders) [5], [6], [7]. Previous work has shown the concurrent application of TDCS can modulate the response to theta burst protocols when TDCS is applied directly over the motor cortex [8]. However, the results of these experiments are difficult to interpret given the bidirectional TMS pulses. Recent studies have suggested that the use a of near-rectangular monophasic pulse that is thought to induce a more “unidirectional” electric field can possibly lead to improved effects of repetitive TMS [9]. As such, here we use a new stimulator capable of delivering unidirectional TMS pulses, and ask whether iTBS using PA pulses is differentially modulated by concurrent directional (PA or AP) TDCS applied across the motor cortex. The experiments were inspired by recent animal studies which have shown that modification of neural membrane potentials with polarising currents can boost or abolish the after-effects of simultaneously applied theta burst stimulation, depending on the site and polarity of stimulation [10], [11]. Although care should be taken when translating slice preparation to *in vivo* studies because of the vast differences in the applied electric fields (10–20 V/mm versus 0.2–0.5 V/mm; [12], [13]) activation of networks may also amplify these effects and be sufficient to modulate likelihood of firing [14]. It is important to note in these experiments that TDCS is applied for a very short time and is concurrent with TMS. This is quite different than experiments that examine homeostatic interactions between TDCS applied for a longer period prior to a second “plasticity” protocol [15], [16], [17].

## Methods

**Fig. 1:**
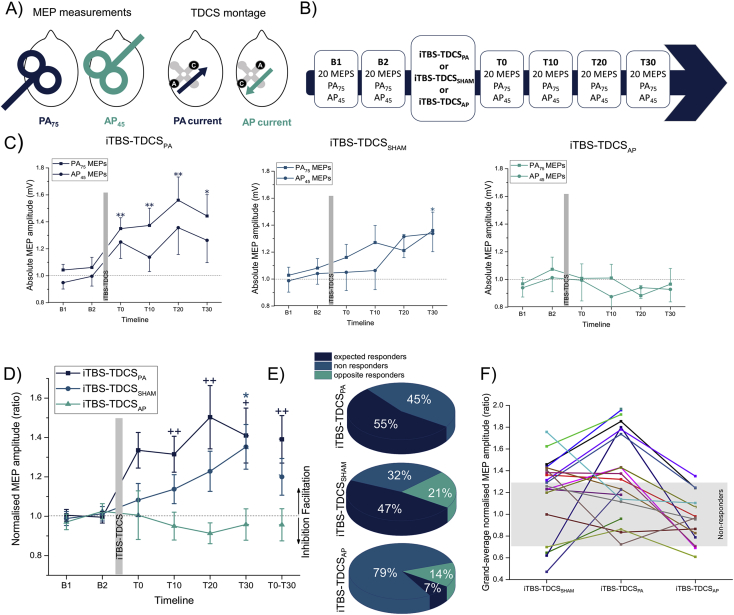
**Effect of iTBS-TDCS on normalised and absolute MEP amplitudes.** A) Left, TMS coil orientations for measurements of PA_75_ and AP_45_ MEPs; Right, TDCS electrode montages. TDCS electrodes were positioned either side of the hotpot and presumed central sulcus. “A” and “C” represent the anode and cathode. TDCS_PA_ refers to an anode-posterior/cathode-anterior montage, while TDCS_AP_ refers cathode-posterior/anode-anterior. Sham was applied using the TDCS_PA_ montage. (B) Timeline of the protocol. MEPs were recorded twice at baseline (B1 and B2) prior to iTBS-TDCS, and at 10 min intervals following iTBS-TDCS. For the iTBS-TDCS_PA_, iTBS-TDCS_AP_ and iTBS-TDCS_SHAM_ sessions PA_75_ MEPs were recorded in 20, 19 and 14 and AP_45_ MEPs were recorded in 15, 15 and 14 individuals due to some individuals having high TMS thresholds and not returning for the final test session. (C) Absolute PA_75_ and AP_45_ MEP amplitudes shown for the three iTBS-TDCS conditions. Paired-sample *t*-tests were computed on absolute MEP average amplitudes to assess each time point versus averaged baseline MEPs (Bonferroni adjusted significance level, p < 0.0125). iTBS-TDCS_PA_ increased MEPs at all time-points, while a statistical trend was found for iTBS-TDCS_SHAM_ at T30 (p = 0.016). *p < 0.0125, **p < 0.002. (D) Averaged PA_75_-AP_45_ MEP amplitudes, normalised to the average of B1 and B2, for the three different conditions. To further assess the time*condition interactions, post-hoc comparisons (Bonferroni adjusted significance level, p < 0.0167) revealed greater MEP amplitudes at T10, T20 and T30 for iTBS-tDCS_PA_, and a statistical trend at T30 iTBS-TDCS_SHAM_, in comparison with iTBS-tDCS_AP._ The grand-average of MEP change (T0-T30) for iTBS-tDCS_PA_ (M = 1.39, N = 19) was significantly greater than iTBS-tDCS_AP_ (M = 0.96, N = 14) (p = 0.001, Cohen's d = 1.33) and tended to be greater than for iTBS-tDCS_SHAM_ (M = 1.20, N = 19) (p = 0.09, Cohen's d = 0.50). A similar trend was observed for iTBS-tDCS_SHAM_ versus iTBS-tDCS_AP_ (p = 0.05, Cohen's d = 0.82). *p < 0.05; + p < 0.017; ++ p < 0.01. (E) Response rates for each condition. Individual average SEM of 20 MEPs from each baseline was computed and averaged across all participants to compute a grand SEM average (±0.15). A significant response to stimulation was considered when it exceeded 95% confidence interval of the SEM (±0.29): opposite responders (OR), <0.71; non-responders (NR), 0.71 > < 1.29; expected responders (ER), >1.29. Compared to iTBS-TDCS_SHAM_, response rates increased 8% and rates of OR decreased from 21 to 0% for iTBS-TDCS_PA_. When the DC current was reversed in iTBS-TDCS_AP_, 79% of individuals were classified as NR and only 7% as ER. (F) Individual grand-average of normalised MEP amplitude (T0-T30). Grey zone represents non-response. Compared to iTBS-TDCS_SHAM_, 11 of 19 participants displayed an increase in MEP amplitudes following iTBS-TDCS_PA_. iTBS-TDCS_AP_ was associated with a decrease in MEP amplitudes compared with iTBS-TDCS_SHAM_ and iTBS-TDCS_PA_ in 12 of 14 participants.

Twenty individuals (12 men; M±SD = 29 ± 8 years; right-handed; no history of neurological or psychiatric disorder) participated in a double-blind randomized crossover protocol, which consisted of two experimental sessions: 1) iTBS + posterior-anterior TDCS (TDCS_PA_), 2) iTBS + sham TDCS (TDCS_SHAM_). Fourteen of those individuals also participated in a third session, where DC current was opposite to TMS: 3) iTBS + anterior-posterior TDCS (TDCS_AP_). All sessions were separated by > five days. TMS was delivered through a figure-of-eight coil (70 mm; Magstim Company Ltd, UK) connected to a cTMS device ([18]: cTMS3; Rogue Resolutions Ltd., UK) over the representation of the right first dorsal interosseous (FDI) muscle. MEPs were recorded via surface electromyography. A neuronavigation system was used to ensure consistent coil positioning (Brainsight, Rogue Resolution Ltd., UK). Following determination of the active motor threshold (AMT), two baseline blocks of 20 MEPs with a ∼1 mV peak-peak amplitude test stimulus (TS_1mV_) were recorded using a near-rectangular monophasic pulse of 45μs duration for antero-posterior (AP_45_), and of 75μs for postero-anterior (PA_75_) stimulation (average PA_75_ TS_1mV_: M = 43% of MSO; average AP_45_ TS_1mV_: M = 87% of MSO) (see Refs. [19], [20]). iTBS was delivered over left M1 using a PA_75_ pulse (80% AMT; 3 pulses at 50 Hz repeated at 5 Hz; 600 stimuli [21]). Electrical current was delivered using a Starstim system (Neuroelectrics, Spain) through a pair of circular 3.14 cm^2^ Ag/AgCl gelled electrodes positioned 3.5 cm posterior and anterior to the TMS hotspot parallel with the orientation of the TMS coil. Stimulation was applied during iTBS for 190s (1 mA, 5sec ramp up/down, sham: 5sec ramp up/down only) (Fig. 1A). Blocks of 20 MEPs (TS_1mV_) using both current directions were recorded for 30min following iTBS-TDCS (Fig. 1B).

## Results

Data are shown mean ± SEM. No differences were obtained among the three sessions for AMT and TS_1mV_ values, and both baseline MEP amplitudes (repeated-measure (RM) ANOVAs; all p > 0.40). To account for unequal group sizes among the three conditions and two coil orientations, a mixed ANOVA (between-group: condition, test pulse; within-group: time) was computed on averaged normalised MEP amplitudes (i.e. divided by the average of B1 and B2). A significant interaction time*condition was obtained (F = 2.93, p = 0.001). Because no significant effect of “test pulse” was obtained (p = 0.86), PA_75_ and AP_45_ MEPs were averaged for further analyses. iTBS-TDCS_PA_ induced facilitation at all time points, and iTBS-TDCS_SHAM_ produced a trend towards facilitation at T30, whereas iTBS-TDCS_AP_ did not induce changes (Fig. 1C). To assess the interaction, a one-way ANOVA (between-subject: condition) was computed on normalised MEPs at each time-point, and the grand-averaged of normalised MEPs (T0-T30). Significant effects were found for T10 to T30 and T0-T30 (all p < 0.05) and LSD post-hoc comparisons were computed (see Fig. 1D for results). Response rates were also computed (see Ref. [22]; Fig. 1E). Individual responses are displayed in Fig. 1F.

## Discussion

Results showed that when the direction of the DC current matched the direction of the electrical field induced by iTBS (posterior-anterior), there was a qualitative increase (19%) in the after-effects of iTBS alone (iTBS-TDCS_SHAM_) that did not reach statistical significance, as well as a slight qualitative increase in “responders” rate. When the direction of the DC current was opposite to the TMS (i.e. anterior-posterior), a significant 24% decrease in cortical excitability changes was observed in comparison with iTBS alone, as well as an important decline in the proportion of “responders” (47%–7%).

Considering the short duration of stimulation (3 min; [23]), it is unlikely that TDCS alone would have produced after-effects on cortical excitability that could interact with iTBS. It seems more likely that TDCS hyperpolarised presynaptic terminals or soma/dendrites of neurons in M1 that were targeted by iTBS, and, as described in animal experiments, this modulated the after-effects of repetitive activity [10], [11], [14]. However, this remains hypothetical as it is not currently possible to be certain which membrane locations might have been affected by TDCS/iTBS in humans since this depends on the details of the electrical field and orientation of neurons, which have yet to be modelled in sufficient detail to address the question. Although we think the results have practical application in using TDCS to boost the effects of rTMS, further detailed work is needed to explore the mechanism.

iTBS did not differentially modulate MEPs recorded using AP_45_ or PA_75_ pulses, which are thought to activate different subsets of neurons when recorded using subthreshold intensities during slight voluntary contraction [1], [2], [3], [24]. This could reflect the fact that our MEPs were recorded at rest using suprathreshold stimulus intensities that recruit multiple neural populations (both early and late indirect waves) [1], [2], [3], reducing the specificity of the directional effect. Alternatively, it could be that this form of unidirectional iTBS equally modulated subsets of neurons recruited by both current directions.

Current results are limited by the fixed order and the smaller sample size for the TDCS_AP_. However, participants were blind to conditions and sessions were separated by at least a week to avoid carry-over/order effects. Arousal changes are also unlikely because of the important scalp activation induced by iTBS compared to TDCS. In addition, because of limitations of our cTMS device we could not test whether AP-iTBS (requiring higher absolute stimulus intensity) would interact similarly with TDCS.

In conclusion, although the present study remains exploratory, our finding suggests that iTBS after-effects can be modulated, and possibly optimised, by concurrent application of directional TDCS.
